# Geographical Patterns of Algal Communities Associated with Different Urban Lakes in China

**DOI:** 10.3390/ijerph17031009

**Published:** 2020-02-05

**Authors:** Shengnan Chen, Huiyan He, Rongrong Zong, Kaiwen Liu, Yutian Miao, Miaomiao Yan, Lei Xu

**Affiliations:** 1 Shaanxi Key Laboratory of Environmental Engineering, Key Laboratory of Northwest Water Resource, Environment and Ecology, MOE, School of Environmental and Municipal Engineering, Xi’an University of Architecture and Technology, Xi’an 710055, China; JiandaHuiyanHe@163.com (H.H.); 18792862643@163.com (R.Z.); kevin_wood1989@163.com (K.L.); miaoyutian728@163.com (Y.M.); Ymmzsj@163.com (M.Y.); Leixugogogo@163.com (L.X.); 2 Institute of Environmental Microbial Technology, Xi’an University of Architecture and Technology, Xi’an 710055, China

**Keywords:** urban lakes, algal bloom, algal community composition, geographical pattern

## Abstract

Urban lakes play an important role in drainage and water storage, regulating urban microclimate conditions, supplying groundwater, and meeting citizens’ recreational needs. However, geographical patterns of algal communities associated with urban lakes from a large scale are still unclear. In the present work, the geographical variation of algal communities and water quality parameters in different urban lakes in China were determined. The water quality parameters were examined in the samples collected from north, central, south, and coastal economic zones in China. The results suggested that significant differences in water quality were observed among different geographical distribution of urban lakes. The highest total phosphorus (TP)(0.21 mg/L) and total nitrogen (TN) (3.84 mg/L) concentrations were found in XinHaiHu (XHH) lake, it also showed highest the nitrate nitrogen (NO_3_^−^-N) (0.39 mg/L),total organic carbon(TOC) (9.77 mg/L), and COD _Mn_ (9.01 mg/L) concentrations among all samples. Environmental and geographic factors also cause large differences in algal cell concentration in different urban lakes, which ranged from 4700 × 10^4^ to 247,800 × 10^4^cell/L. Through light microscopy, 6 phyla were identified, which includes Chlorophyta, Bacillariophyta, Cyanophyta, Dinophyta, Euglenophyta, and Cryptophyta. Meanwhile, the heat map with the total 63 algal community composition at the genus level profile different urban lakes community structures are clearly distinguishable. Further analyses showed that the dominant genera were *Limnothrix* sp., *Synedra* sp., *Cyclotella* sp., *Nephrocytium* sp., *Melosira* sp., and *Scenedesmus* sp. among all samples. The integrated network analysis indicated that the highly connected taxa (hub) were *Fragilaria* sp., *Scenedesmus* sp., and *Stephanodiscus* sp. The water quality parameters of NO_3_^−^-N and NH_4_^+^-N had significant impacts on the structural composition of the algal community. Additionally, RDA further revealed distinct algal communities in the different urban lakes, and were influenced by NO_2_^−^-N, Fe, and algal cell concentrations. In summary, these results demonstrate that the pattern of algal communities are highly correlated with geographic location and water quality on a large scale, and these results also give us further understanding of the complex algal communities and effectively managing eutrophication of urban lakes.

## 1. Introduction

With the rapid development of industrialization and the acceleration of urbanization, industrial water and drinking water have increased sharply [1]. Rivers and lakes in many cities have become long-term retention places for wastewater [2]. Urban lakes have the characteristics of drainage and water storage, regulating urban microclimate conditions, and meeting citizens’ recreational needs [3,4,5]. Meanwhile, most of the urban landscape water bodies are static or semi-closed slow-flowing, which have the characteristics of small water environment capacity, relatively fragile ecosystem, and limited self-purification [6]. When the temperature is high, the water body rich in nitrogen and phosphorus nutrients is eutrophic, then algal blooms are formed. Abnormal reproduction of algae can cause odor and color problems, and destroy the ecological balance of water bodies [7]. In the past few decades, researchers have focused on the geographical distribution of microorganisms in oceans and temperate lakes, but relatively few studies have been done on urban lakes [8,9]. In recent years, the eutrophication of urban lakes, especially the outbreak of algae, is a potential huge hazard to people’s lives due to its special geographical location and can seriously affect the development and utilization of water resources [10]. Thus, studies on the fundamental features of urban lake ecosystems are of great importance because they can provide a theoretical basis for our better recovery and management of urban lakes.

From the ecological point of view, changes in algal community diversity and composition are an important indicator for evaluating the water quality status and changing trend of rivers and lakes [11,12]. From an ecological point of view, the water environmental factors directly affect the population or community type of algal structural feature [13], on the other hand, the individual, population, or community of algae change can objectively reflect the changing discipline of water quality [14].Therefore, studying the relationship between algae and lake ecosystems is of great significance for the management and restoration of polluted water bodies. In recent years, efforts have been made to determine the algal community structures of seasonal and spatial variations [15]. However, few studies have addressed the characteristics of algal community structure in different urban lakes and the correlation of community structure with water quality in a large scale.

In freshwater ecosystems, parameters of urban polluted water such as pH, total nitrogen, total phosphorus, and total organic carbon are important indicators for studying the relationship between geographical patterns of different cities and algal communities’ structures [16,17,18,19,20]. Some researchers in the past have explored microbial community diversity in drinking water reservoirs [21], polluted rivers [22], and drinking water pipes [23]. In lake ecosystems, many reports have focused on revealing water temperature and nutrients and other important environmental factors affecting the growth of phytoplankton [24,25]. For instance, Habib et al. [26] studied the effects of seasonal changes and physical chemical factors on Locus Lomond and suggested that dissolved oxygen and nutrients were significantly related to the phytoplankton community distributions. However, few people probe the relationship between environmental factors and algal community in urban lakes under different spatial locations. In addition, only a limited number of studies have focused on interaction between algae under different spatial and environment condition, especially from a network perspective.

Recently, network has been widely used to explore the microbe co-occurrences and the environmental conditions that correlate with these eukaryotic plankton co-occurrence patterns [27,28,29,30]. Network analysis technology is a systematic analysis method for analyzing the intrinsic interaction between ecosystems by basic stochastic matrix theory. It is used to synthesize and characterize the molecular ecological network of microbial aggregation, providing a reliable method to understand of the potential interaction of complex microbial clusters [31]. In the network, each element (biological or genetic) can be described as a node, and the relationship between them can be described as a directed or undirected edge [32,33]. In our previous research, Zhang et al. [34] used co-occurrence network patterns to reveal different community interactions between aerobic/anoxic/aerobic and oxidation ditch systems in 18 geographically distributed wastewater treatment plants in nine provinces of China. Recently, Liu et al. [28] also used eukaryotic plankton co-occurrence networks and found that the cyanobacterial biomass cycle was remarkably linked to eukaryotes in two subtropical reservoirs over a 6-year period. Moreover, they further constructed four subnetworks based on distinct eukaryotic community succession periods and showed that the eukaryotic co-occurrence patterns were varied significantly correlating with cyanobacterial biomass. However, co-occurring networks associated with the algal coexistence at different levels of whole communities in different urban lakes have not been investigated.

To this end, the specific objectives of this work were to (1) determine the physiochemical parameters of urban lakes water quality, (2) investigate the algal cell morphology and community based on microscope, and (3) assess the relationship among water quality, algal concentration, and community structure, meanwhile, unveil algal community co-occurrence interactions in urban lakes distributed across a wide range of geographical locations. The results from this work will have certain reference significance for understanding the complex algal communities and effectively managing eutrophication of urban lakes.

## 2. Materials and Methods

### 2.1. Site Description and Field Sampling

**Figure 1:**
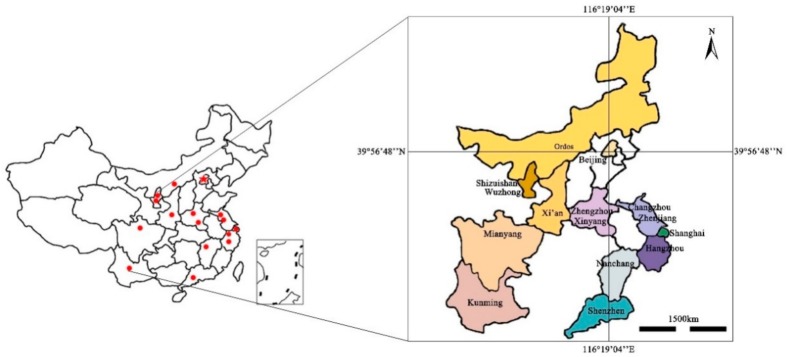
Geographical location of 16 urban lakes sampled in China.

To compare the algal community composition of urban lakes from various regions of China, sampling points were selected from different provinces located from the south to north (25°04′10″ N–39°56′48″ N) and east to west areas (121°25′23″ E–102°42′53″ E). As shown in Figure 1, the urban lakes were located in different geographic locations (Shaanxi, Sichuan, Henan, Jiangsu, Zhejiang, Jiangxi, Yunnan and Guangdong province, Inner Mongolia and Ningxia autonomous region, Shanghai and Beijing municipalities). The 16urban lakes are TieXi (TX), XinHaiHu (XHH), JinJi (JJ), ChangLe (CL), XiangShan (XS), AiXi (AX), HuiLongShan (HLS), GaoTie (GT), JinSha (JS), XiLiu (XL), ZiZhuYuan (ZZY), GuiLong (GL), ZhuZhai (ZZ), ZhongShan (ZS), West lake (WL), and YuNv (YN) (Table S1). 

The detailed information about urban lakes is listed in Table 1. The sampling process was undertaken in October 2018 and collected from the surface water of urban lakes. Water samples were collected at a depth of 0.5 m in each urban lake with sterilized polypropylene containers [35]. One part of the water sample was used to determine the water physicochemical characteristics and algal cell concentration. Another part of the water sample was used for algal morphology and community diversity examination.

### 2.2. Water Physicochemical Analysis

To determine the water quality parameters, the pH, nitrite nitrogen (NO_2_^−^-N), nitrate nitrogen (NO_3_^−^-N), ammonia nitrogen (NH_4_^+^-N), total nitrogen (TN), total phosphorus (TP), total organic carbon (TOC), permanganate index (COD _Mn_), Fe, and Mn concentrations were measured. The pH was measured using a pH meter (Hach, USA) in the field. NO_2_^−^-N, NO_3_^−^-N, NH_4_^+^-N, and TN concentrations were determined using a flow injection analyzer (FIA) (Seal Analytical AA3, Norderstedt, Germany) based on a previously described method [35]. TP was measured using a spectrophotometer (DR6000, Hach, USA). TOC was measured using a TOC analyzer (TOC-L CPN, Japan). COD _Mn_ was examined using a spectrophotometer (UV-mini 1240, Shimadzu, Japan) [36,37]. Fe and Mn concentrations were measured using Inductively Coupled Plasma Mass Spectrometry (ICP-MS). 

### 2.3. Algal Cell Concentration and Community

To measure the algal cell concentration, 500 mL of surface water samples through 0.45μm polycarbonate membrane (47mm diameter, Millipore, USA). The algae enriched on the 0.45 μm polycarbonate membrane were concentrated to a final volume of 10 mL, and 1% Lugol’s iodine solution was added there. Algae were identified to the phyla/ genus level and counted using a microscope (Olympus BX51, Japan) following Shen et al. [38], Zhang et al. [39], and Hu et al. [40] and reported in terms of × 10^4^ cells per liter. To further demonstrate the variety of algal morphology, 100 μL of algae fluid was fixed by the above method, dropped on a glass slide, a picture was taken under a 400 × microscope (50I, Nikon, Japan). Typical algal pictures were selected for display in this study. The assays were performed in triplicate. 

### 2.4. Network Construction

The integrated network was generated using a visualized Gephi platform (version 0.9.2) based on samples from 16 different urban lakes, which was constructed for strong (*r* ≥ |0.6|) and statistically significant (*p*-value < 0.05) correlations incorporated into network analyses [41,42]. A total of 52 identified genera of algae and 10 environmental factors (pH, TN, NO_3_^−^-N, NO_2_^−^-N, NH_4_^+^-N, TP, TOC, COD _Mn_, Fe, Mn) were included in the networks. For modular analysis, gephi employed the Louvain method developed by Blondel et al. [43]. For modular analysis, the size and color of the nodes indicate the number of samples and classification, respectively. The thickness and color of the line connecting two nodes (i.e., edge) represent the Spearman’s correlation coefficient (*r*) and the positive or negative correlation, respectively. Gephi was also applied to determine node-level topology properties (i.e., degree, betweenness, and closeness centralities). Degree centrality is the number of directly connected nodes. Betweenness centrality refers to the number of shortest paths going through a node. Closeness centralities are the sum of the shortest distances from one node to other nodes [44]. Many topological parameters (e.g., the number of nodes and edges, average path length, network diameter, average degree, graph density, clustering coefficient, and modularity index) were calculated using the igraph package in R [45].

### 2.5. Statistical Analysis

To compare the mean value of water quality parameters and algal cell concentration in different urban lakes, statistical analyses were performed using one-way factorial analysis of variance (ANOVA) followed by a Tukey HSD post-hoctest using SPSS (version 17.0, SPSS Inc, Chicago, IL, USA). The distribution of algae at the phyla level in 16 different urban lakes was visualized by using Circos (version 0.69, http://circos.ca/). Heat map profiles were performed using R software to compare the algal community structure at the genus level(version 3.2.3) [46]. A correspondence analysis revealed that the length of the first axis was less than three [47]. The interrelation between the water quality and water algal communities of different urban lake samples were measured using multivariate correlation analysis (redundancy analysis, RDA), which was performed using the Canoco software package for Windows (version 4.5) (Ithaca, New York, USA) with Monte Carlo permutation tests (999 permutations). The graphics were generated in Cano Draw (version 3.10) for Windows [47].

## 3. Results and Discussion

### 3.1. Water Quality Parameters

The geographic locations of the studied urban lakes are summarized in Table 1. All of the 16 different urban lakes physical parameters (pH) and nutrient concentrations (e.g., TP, TN, NO_3_^−^-N, NO_2_^−^-N, NH_4_^+^-N, TOC, COD _Mn_, Fe, and Mn) are summarized in Table 2. Water quality characteristics were distinct among the different urban lakes’ geographical locations. The pH values ranged from 7.27 in GL lake to 9.30 in TX lake (*F* = 94.319, *p* < 0.001). The highest TP (0.21 mg/L), NO_3_^−^-N (0.39 mg/L),and TN (3.84 mg/L) concentrations were found in XHH lake (*F* = 2106.073, *p* < 0.001; *F* = 501.578, *p* < 0.001; *F* = 1138.293, *p* < 0.001), it also showed highest the TOC and COD _Mn_ concentrations among the samples. The lowest TOC (1.09 mg/L) concentrations were observed in CL lake (*F* = 270.402, *p* < 0.001). The NH_4_^+^-N concentrations varied from 0.01 to0.50 mg/L (*F* = 452.529, *p* < 0.001). The COD _Mn_ concentration ranged from 4.21 in GT lake to 9.01 in XHH lake (*F* = 114.866, *p* < 0.001). It is worth noting that the Fe and Mn concentrations were low in all urban lakes (*F* = 22.044, *p* < 0.001; *F* = 6.267, *p* < 0.001). In addition, both JS lake and WL lakes are located in Hangzhou, but the TN concentrations in JS lake was approximately two times higher than that of WL lake. Similarly, Yang et al. [48] found that the 16 urban water sampling points pH values were between 6.9 and 9.8 in western China, and the distance between the sampling lakes was 9–2027 km. In addition, a large number of previous studies have shown that spatial distance can create spatial differences in water quality characteristics [49]. XXH lake is the largest wetland park in the northern part of Shizuishan, where the area and ecological environment are exposed to human activities and industrial and agricultural development. Human activities are bound to cause pollution of urban water bodies, and a large amount of domestic garbage and heavy metals enter the water bodies directly or through release of sediment indirectly. Xiong et al. [49] suggested that human activities and climate change affect water quality in two agricultural catchments in Finland, the result is consistent with this study. Furthermore, XXH lake is composed of lake wetlands, barren sandy land, fishing ponds, and farmland. There are three pulverized coal plants around the lake, which are the main pollution sources of XXH lake. Aquaculture and agricultural wastewater cause a large amount of nitrogen and phosphorus nutrients to be input into wetland water bodies [50,51]. In addition, the surface of fly ash contains a certain amount of adsorbed nitrate nitrogen which further aggravates the accumulation of nitrogen in water bodies. Previous research reported that only 20%–25% of the protein in the feed was generally absorbed by fish, and most of the rest was discharged to the water body in the form of ammonia or organic nitrogen, causing eutrophication of the surrounding water bodies [52,53]. The pollution of agricultural wastewater mainly comes from abuse of organic fertilizer by farmers. Green et al. [54] stated that due to the long-term transport of N elements caused by the large amount of fertilizer input in the corn planting area, the nitrate and nitrite content of the water body in Edwards changed significantly. Another important reason for the high nitrogen content in the water body of XHH lake was that nitrogen adsorbed on the surface of fly ash accumulated in the fly ash plant for a long time and entered the water body under the wash of rainwater [55]. WL lake is located in the middle of Hangzhou, Zhejiang Province, and is a famous urban tourist lake in China. After 1960, the massive discharge of urban sewage led to the eutrophication of the West Lake intensively. In 2002 and 2006, Hangzhou city launched the West Lake Westward Project and the aquatic vegetation ecological restoration project, respectively. The water nutrient status gradually changed from eutrophic to medium nutrient level. The result showed that water quality of urban lakes is different, when it has similar locations and climatic conditions. Historical environment changes and human disturbances can also affect the water environment. Andersson et al. [56] also concluded that the spatial difference of urban lakes water quality is affected by environmental conditions and historical events. Moreover, Jiang et al. [17] found that the concentrations of PO_4_^3−^-P, TP, NH_4_^+^-N, and NO_2_^−^-N in western rivers around Lake Chaohu were much higher than those in other samples of lake Chaohu. Simultaneously, Shang et al. [57] found that the eutrophication state of the lake Chaohu western part is more serious than that of the eastern part, mainly because the former is the final place of industrial and municipal wastewater from Hefei City, the capital of Anhui province. Therefore, the water quality of urban lakes may be affected by geographical environment factors, industrial and agricultural wastewater, human activity, and historical environment changes. 

### 3.2. Algal Cell Concentration

**Figure 2:**
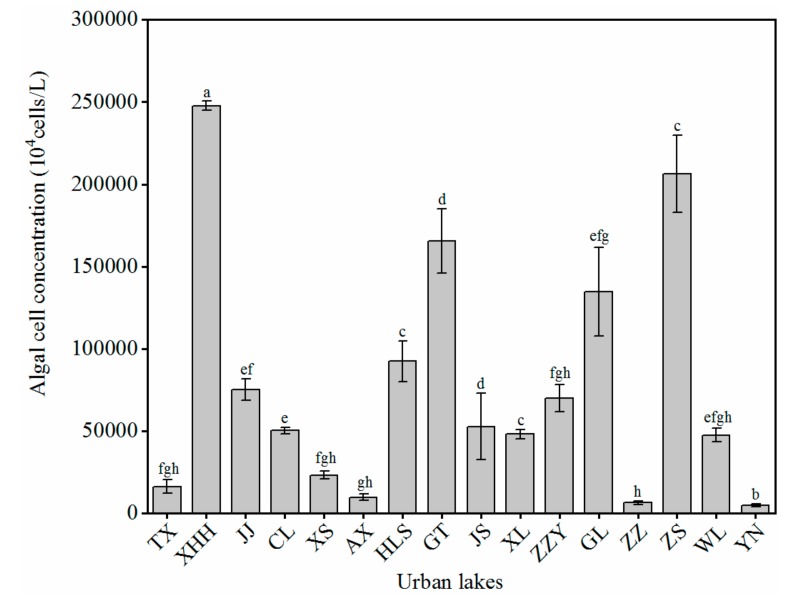
Algal cell concentration of 16 different urban lakes in October 2018. Bars with different upper letters are significantly different at 0.01 levels. Error bars represent standard deviations (*n* = 3).

In the present study, we investigated the algal cell concentrations in 16 urban lakes, collected at various sites from different regions of north, central, south, and coastal economic zones in China (Figure 2). The algal cell concentration was high in XXH lake (247,800 × 10^4^ cell/L) and ZS lake (206,300 × 10^4^ cell/L). These values were much higher than other sample sites. XXH lake is located in Dawukou district, Shizuishan City, a newly developed industrial city in Yinbei. With the development of urbanization, human domestic waste, domestic sewage, and industrial wastewater in cities and towns, flow into surface water in the form of surface runoff. High input of nutrients such as nitrogen and phosphorus caused an algae outbreak in XHH lake, previous numerous reports also support this point of view [58]. In addition, nutrient enrichment facilitates algal outbreaks in eutrophic shallow lakes. The N plus P amendment promoted higher biomass of the planktonic microbial community, and the dual addition of NH_4_^+^+PO_4_ yielded the highest chlorophyll a concentration, as found by Dodd in other freshwater lakes [18]. Compared with XHH lake, the concentration of nitrogen and phosphorus in ZS lake is relatively low, but ZS lake is located in Shenzhen, which has a subtropical maritime climate with warm and humid seasons, abundant rain, and abundant sunshine. Temperature has a certain influence on the abundance and biomass of algae. When the water temperature reaches the optimum temperature for algal growth, the primary productivity begins to rise rapidly. The conclusion is consistent with Huber’s findings; Huber et al. [59] suggested that nutrient loading and winter temperature influence the timing of the phytoplankton spring bloom. The concentration of algal cells in the urban lakes of JS lake (52,700 × 10^4^ cell/L), WL (47,500 × 10^4^ cell/L), GT lake (165,400 × 10^4^ cell/L), HLS lake (92,400 × 10^4^ cell/L), and ZZ lake (6300 × 10^4^ cell/L) are relatively high. Those five urban lakes are located in the Jiangsu, Zhejiang, and Shanghai regions in the eastern Yangtze River Delta of China. Jiangsu, Zhejiang, and Shanghai are in a subtropical monsoon climate with abundant sunshine and rainfall. There are strong storms and rains in these coastal areas during the summer. Rainfall can dilute pollutants in urban waters, but also carry nutrients into the water. Heavy rain increased the nutrient content and further affected the algal outbreak in the water body. Similarly, Greenaway et al. [60] investigatedthe effects of groundwater and rainfall on the ambient concentrations of inorganic nitrogen and phosphorus in the coastal waters of Discovery Bay, Jamaica. The results documented that heavy, widespread rainfall events significantly increased the concentration of NO_3_^−^-N, and in severe cases elevated NO_3_^−^-N concentrations were sustained for several months. Based on the above comparative analysis, we found that the biomass of algae reveals a contrast change on a spatial scale (Figure 2). Nutrition, rainfall, and temperature are the main factors that cause significant differences in algal cell concentration in different urban lakes.

### 3.3. Geographical Patterns of Algal Community Composition and Typical Cell Morphology

**Figure 3:**
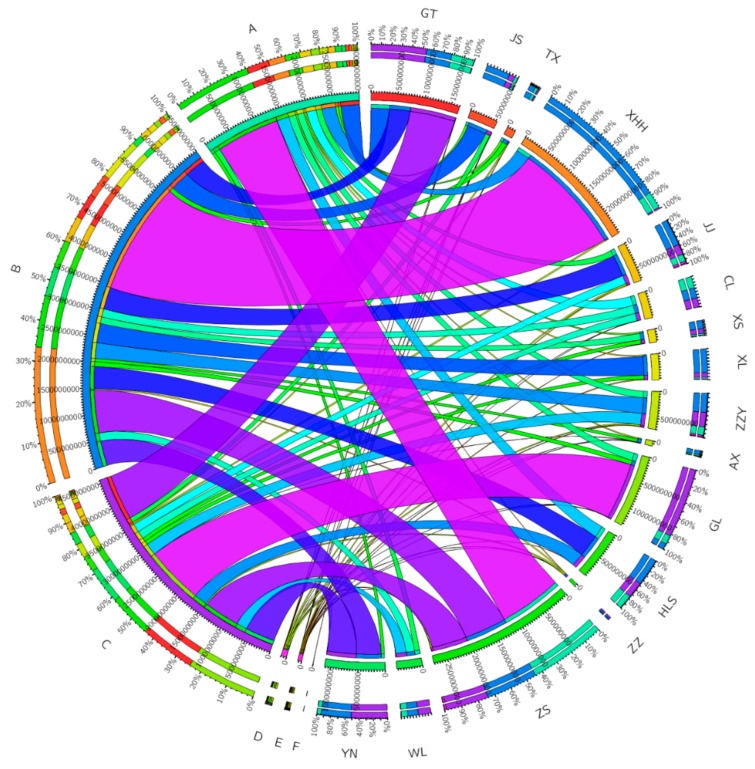
Circos representation of algal community at phyla level. The bands in the same urban lakes with different colors demonstratethe source of different phyla. (**A**) Chlorophyta, (**B**) Bacillariophyta, (**C**) Cyanophyta, (**D**) Dinophyta, (**E**) Euglenophyta, (**F**) Cryptophyta. The data were visualized via Circos software (http://circos.ca/).

A total of six phyla were identified from 16 urban lakes, with qualitative and quantitative identification of algae including Chlorophyta, Bacillariophyta, Cyanophyta, Dinophyta, Euglenophyta, and Cryptophyta. Across all samples from 16 urban lakes, half of the urban lakes were dominated by Bacillariophyta (accounting for 88.3% in XHH lake, 76.8% in XL lake,71.2% in JS lake,65.0% in AX lake, 56.6% in JJ lake, 54.9% in TX lake, 53.3% in XS lake, 43.4% in ZZY lake, and 47.1% in HLS lake), followed by Cyanophyta (accounting for 82.4% in GLlake,53.3% in GTlake, 51.3% in YN lake, 43.1% in ZZ lake, 43.0% in WL),last by Chlorophyta (accounting for 37.2% in CLlake, and 44.3% in ZS lake) (Figure 3). Similar to our conclusion, Yang et al. [61] previously investigated the algal community characteristics by optical microscopy of 11 typical subtropical reservoirs in southeast Fujian, the result revealed that algal communities varied strongly across study reservoirs and the dominant phyla were Chlorophyta, Cyanophyta, Bacillariophyta, and Chrysophyta, which accounted for 92.01% of the mean relative abundance. These studies indicate that the algal community is affected by the local environment and geographical distribution, and which are the inherent physiological factors.

**Figure 4:**
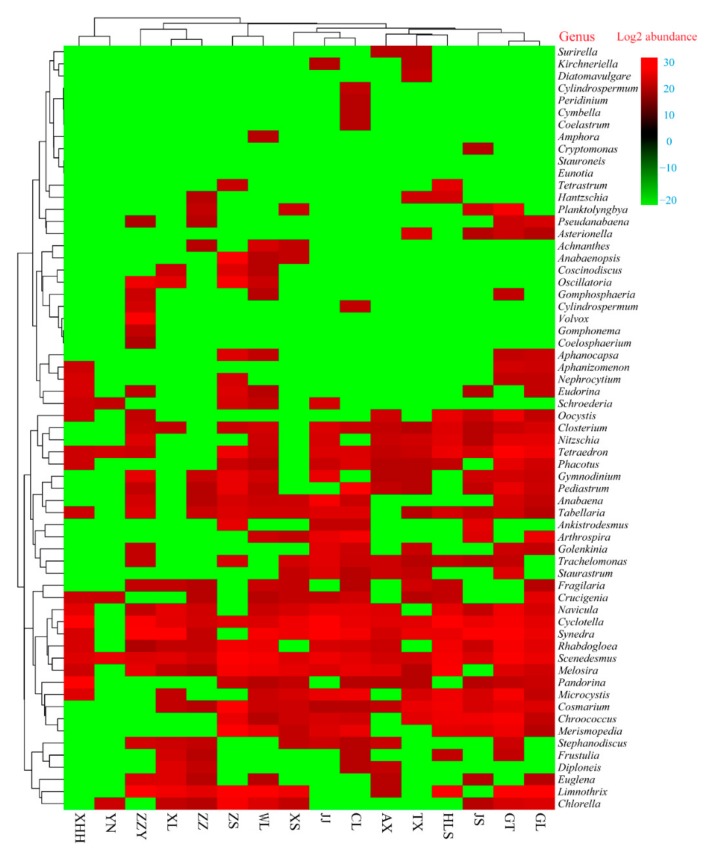
Heat map profile showing the algal community at the genus level in 16 different urban lakes. Green colors indicate lower abundance, red colors indicate higher abundance. GL, GT, JS, HLS, TX, AX, CL, JJ, XS, WL, ZS, ZZ, XL, ZZY, YN, and XHH represent GuiLong, GaoTie, JinSha, HuiLongShan, TieXi, AiXi, ChangLe, JinJi, XiangShan, WestLake, ZhongShan, ZhuZhai, XiLiu, ZiZhuYuan, YuNv, and XinHaiHu urban lakes, respectively.

**Figure 5:**
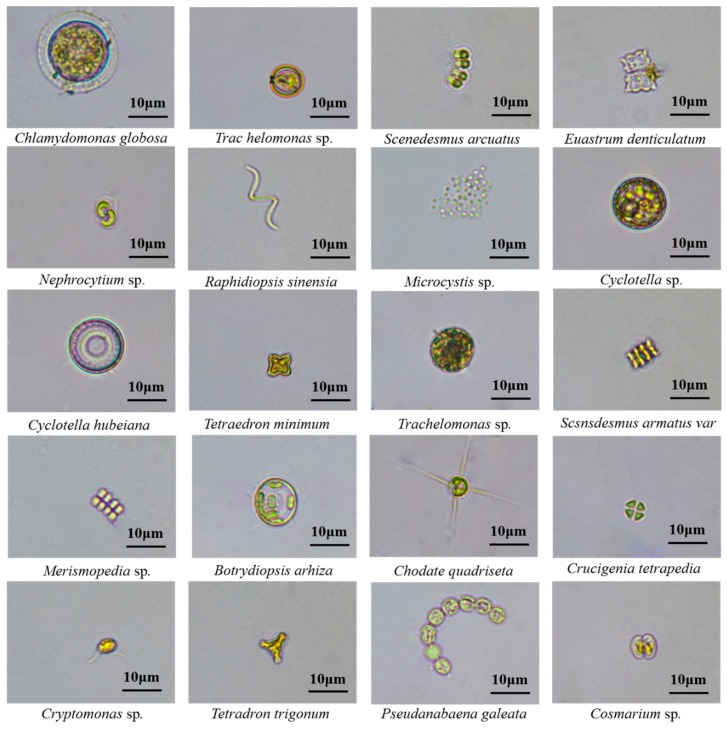
Microscopic images of typical algae in 16 different urban lakes.

To further establish a detailed view on the algal community, as shown in Figure 4, a heat map profile with the total 63 algal community composition at the genus levels was drawn. Generally, the heat map indicated that algal communities in each urban lake were unique, and revealed that the algal communities in 16 urban lakes were more diverse and different. Some common algal morphologies during algae outbreaks are shown in Figure 5. For instance, *Limnothrix* sp. were the dominant genera in GL lake (29%), XS lake (20%), ZZY lake (23%), GL lake (77%), ZZ lake (35%), WL lake (25%), and YN lake (44%), *Synedra* sp. were the dominant genera in JS lake (64%), TX lake (22%), and XL lake (61%), *Cyclotella* sp. were the dominant genera in XXH lake (86%), JJ lake (32%), and AX lake (19%), *Nephrocytium* sp., *Melosira* sp., and *Scenedesmus* sp. were the dominant genera in CLlake (25%), AX lake (19%), and ZS lake (27%), respectively. Notably, GT lake and WL lake were the abundant genera in all samples. In the algal ecological classification, the above dominant algal species belong to Cyanophyta, Bacillariophyta, and Chlorophyta. Similar studies have suggested that the superiority of the genus *Limnothrix* sp. in Lake Kastoria, at the same time, high population densities over the winter and before the development of daphnia may be the main reason [62,63]. Besides, numerous previous studies have reported that algal bloom may be strongly correlated with Cyanobacteria, which was one of the most visible symptoms of eutrophication, particularly in warm, dry summers [63,64,65]. *Synedra* sp. is usually the dominant genus in the low temperature season [66,67]. For example, Bracht et al. [68] studied diatom deposits in Lake Crevice, Yellowstone National Park, USA, and found that in the long and cold spring, *Synedra* sp. was one of the dominant diatoms in the sediment. Cyclotella, one of the common diatoms in fresh water, is also the dominant algal species that cause algal blooms [69]. More importantly, we found that the spatial patterns were not only closely related climate environment but also to algal community structure characteristics.

### 3.4. Co-Occurrence Network of Algae

**Figure 6:**
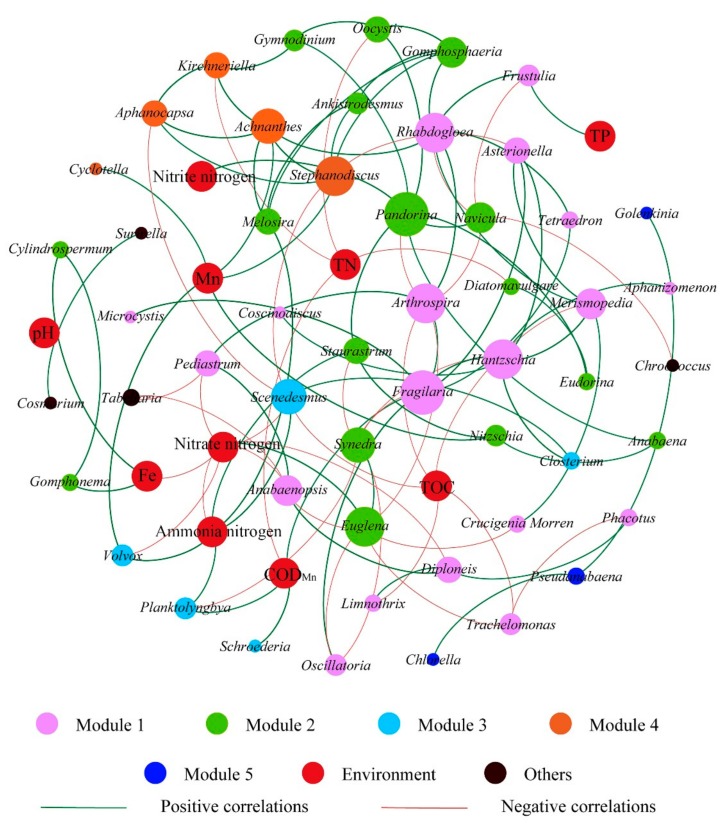
Network analysis revealing the modular associations between algal communities and environmental factors from 16 different urban lake samples. A connection stands for a strong (Spar CC |*r*| > 0.6) and significant (*p*-value < 0.05) correlation. The nodes are colored according to modularity class. The size of each node represents value of betweenness centrality.

The co-occurring network has been successfully applied to infer microbial co-occurrence patterns in soil microbial communities [70], bacterial wastewater treatment plants [34], urban lakes denitrifying bacteria [71], and subtropical reservoirs eukaryotic plankton [28]. In this study, an integrated network was used to explore modular associations between algal taxa (i.e., abundant genera) and environmental factors in 16 geographically distributed urban lakes (see Figure 6 and Supplementary Materials), additionally, the properties of the integrated networks are summarized in Table 3. The integrated network is comprised of 62 nodes and 108 edges, with an average number of clustering coefficient of 0.209 and path length of 3.231. Meanwhile, all nodes in the network can be divided into five main modules, which accounted for 95.8%of the whole networks. Among the 10 water quality parameters of module V, the two parameters of NO_3_^−^-N and NH_4_^+^-N are worthy of attention. NO_3_^−^-N and NH_4_^+^-N are associated with the largest number of nodes and mainly negatively and positively correlated with other nodes, respectively. Our results indicated that the algae biomass cycle in 16different urban lakes were strongly correlated with the algal community composition compared to other physical and chemical factors. However, the physical and chemical factors were significantly correlated with the algae biomass, and could indirectly influence the algal community composition [72]. Co-occurrence correlation analysis shows that the algal species are predominantly positive correlations. This suggests that mature microbial communities, niche separation, and less competition between algal species exist in the urban lakes. Algae species had the highest centrality values, which indicate their important role in the algal community. By analyzing the network, we found that keystone species belonged to *Fragilaria* sp., *Scenedesmus* sp., and *Stephanodiscus* sp., which as keystone taxa play an important role in maintaining network structure, compared with other taxa in the network [73]. Once keystone taxa disappear, the network may disassemble [28], keystone taxa play an important role in maintaining the stability of the ecosystem [74]. Therefore, co-occurrence network is a powerful technique for giving insights into the organization of algal communities and studying interactions that occur between algal communities, such as competition and resonance.

### 3.5. Relationship between Algal Communities and Water Quality

**Figure 7:**
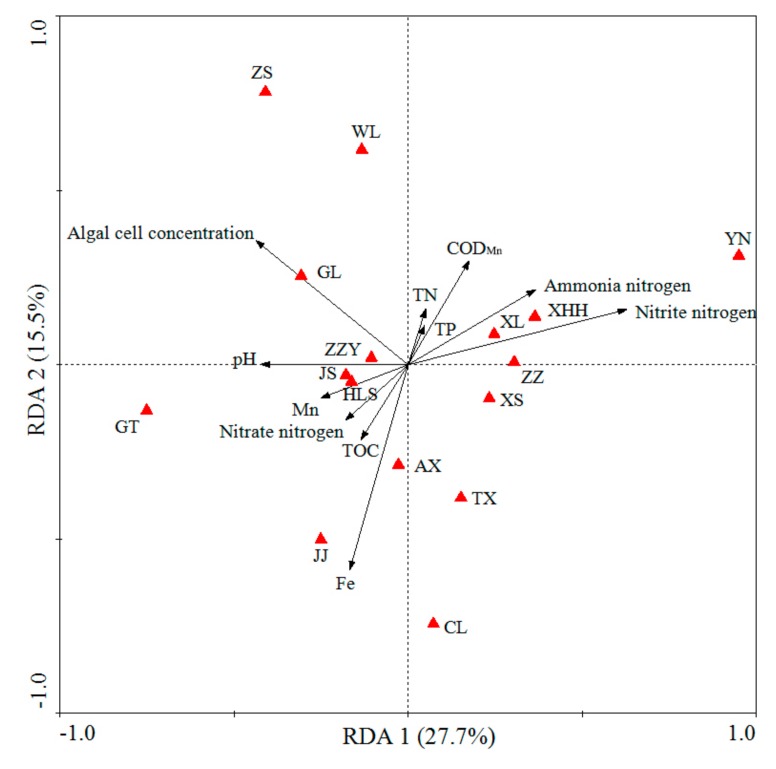
Redundancy analysis (RDA) of water algal communities in 16 geographically distributed urban lakes. Red triangles represent sampling points. For algal community, RDA1 explained 27.7% and RDA2 explained 15.5% of the total variance. The factors of the water quality data are represented by arrows (TN = total nitrogen; TP = total phosphorus; TOC = total organic carbon). Water quality parameters that significantly correlated with algal community diversity are shown.

To identify the relationship between algal communities of the different urban lakes and water parameters, redundancy analysis (RDA) which was consistent with the heat map was conducted. RDA1 andRDA2 explained 27.7%and 15.5% of total variances, respectively(as shown in Figure 7). The first axis RDA1 was positively correlated with water NO_2_^−^-N, NH_4_^+^-N, COD _Mn_, TN, and TP, but negatively correlated with Mn, NO_3_^−^-N, TOC, and Fe. The second axis of RDA2 was positively correlated with water algal cell concentration and pH. Monte Carlo permutation tests also revealed that Fe, NO_2_^−^-N, and algal cell number were significantly correlated with the changes in the algal composition. The RDA diagram showed that JS lake, HLS lake, GT lake, JJ lake, and AX lake were located in the third quadrant, whereas CL lake, TX lake, and XS lake were located in the fourth quadrant. RDA results revealed the differences among the algal communities in the 16 different urban lakes, which had diverse physic-chemical water properties. An increasing number of studies have reported that nitrogen and phosphorus have a decisive effect on the growth of algae [75]. Nitrogen is not only a substance necessary for algal growth and metabolism, but also one of the main constituent elements of proteins and nucleic acids in the algae. In addition, studies have also proven that nitrogen in different forms could affect the absorption and utilization of algae [76,77]. Phosphorus is not only the main component of nucleic acids, proteins, and phospholipids, but also a substance necessary for the synthesis of chlorophyll. Therefore, phosphorus can further affect algal growth by affecting algae photosynthesis [78]. COD _Mn_ is related to the state of organic pollution in water bodies, and is often used as an index to measure the organic matter content in lakes, reservoirs, and other water bodies. Qiu et al. [79] found that COD _Mn_ has a significant effect on the concentration of algae and its community dynamic distribution in the sand lake, which is consistent with our research results. The ecological factor of pH is closely related to algal growth, and different algae have certain adaptation ranges [80], Furthermore, the changes of pH can increase or decrease the release of phosphorus from Fe and Al compounds to indirectly affect algal community structure [81]. For example, Rai et al. [82] investigated that the influence of pH on the growth of three species of cyanobacteria, two species of diatoms, and one species of planktonic green algae, and the result showed that six species of marine phytoplankton preferred near neutral to alkaline pH. Therefore, pH has an important influence on the composition and distribution of algal species. These results give us a better understanding of the relationship between algal community structure and water quality.

## 4. Conclusions

In summary, our results demonstrated that the water quality in 16urban lakes was significantly different; meanwhile, the pattern of algal communities was highly correlated with geographic location and water quality. By light microscopy analysis, a total of six phyla and 63 genera were identified, of which Bacillariophyta, Cyanophyta, and Chlorophyta were dominant. Our results also indicated that the dominant genera were *Limnothrix* sp., *Synedra* sp., *Cyclotella* sp., *Nephrocytium* sp., *Melosira* sp., and *Scenedesmus* sp. Network analysis suggested that the highly connected taxa (hub) were *Fragilaria* sp., *Scenedesmus* sp., and *Stephanodiscus* sp. Meanwhile, the water quality parameters of NO_3_^−^-N and NH_4_^+^-N had a significant impact on the structural composition of the algal community. RDA revealed that algal communities were greatly distinct in 16urban lakes and they were mainly affected by geographic location, NO_2_^−^-N, Fe, and algal cell concentration. Therefore, our studies of algal communities at a large spatial scale contribute to more effective governance and restoration of ecosystem functions in eutrophic urban lakes. 

## Figures and Tables

**Table 1:** The 16 urban lakes located in different areas of China.

Urban Lakes	Provinces	Cities	Latitude	Longitude	Average Monthly Temperature (°C)	Surface Area(m^2^)	Urban Population	Built Year
TieXi (TX)	Inner Mongolia	Ordos	39°49′16″ N	109°58′07″ E	15.3	1.7 × 10^4^	2.0 × 10^6^	2005
XinHaiHu (XHH)	Ningxia	Shizuishan	38°59′32″ N	106°24′22″ E	16.7	2.0 × 10^7^	7.9 × 10^5^	2004
JinJi (JJ)	Ningxia	Wuzhong	37°56′10″ N	106°08′36″ E	17.3	2.0 × 10^8^	1.3 × 10^6^	Qin and Han Dynasties
ChangLe (CL)	Shaanxi	Xi’an	34°16′04″ N	109°00′00″ E	24.7	2.2 × 10^5^	8.8 × 10^6^	1956
XiangShan (XS)	Henan	Xinyang	31°34′27″ N	114°55′00″ E	26.0	1.1 × 10^7^	6.4 × 10^6^	1969
AiXi (AX)	Jiangxi	Nanchang	28°42′56″ N	115°59′21″ E	29.7	4.5 × 10^6^	5.5 × 10^7^	2007
HuiLongShan (HLS)	Jiangsu	Zhenjiang	32°09′24″ N	119°27′07″ E	22.0	1.3 × 10^7^	3.1 × 10^6^	1977
GaoTie (GT)	Jiangsu	Changzhou	31°51′21″ N	119°58′07″ E	26.3	1.0 × 10^5^	3.8 × 10^6^	2017
JinSha (JS)	Zhejiang	Hangzhou	30°18′52″ N	120°20′00″ E	28.3	3.1 × 10^4^	9.2 × 10^6^	2018
XiLiu (XL)	Henan	Zhengzhou	34°46′00″ N	113°34′36″ E	28.7	4.6 × 10^8^	1.1 × 10^7^	2012
ZiZhuYuan (ZZY)	Beijing	Beijing	39°56′48″ N	116°19′04″ E	23.3	1.9 × 10^5^	2.2 × 10^7^	1953
GuiLong (GL)	Yunnan	Kunming	25°04′10″ N	102°42′53″ E	24.0	1.6 × 10^5^	6.7 × 10^6^	2006
ZhuZhai (ZZ)	Shanghai	Shanghai	31°12′53″ N	121°17′36″ E	27.3	3.5 × 10^4^	1.4 × 10^7^	2004
ZhongShan (ZS)	Shenzhen	Shenzhen	31°13′27″ N	121°25′23″ E	33.3	3.5 × 10^4^	1.1 × 10^7^	2004
West lake (WL)	Zhejiang	Hangzhou	30°13′14″ N	120°06′30″ E	29.0	6.4 × 10^6^	9.2 × 10^6^	Qin and Han Dynasties
Yunv (YN)	Sichuan	Mianyang	31°29′54″ N	104°44′14″ E	25.3	2.4 × 10^5^	5.4 × 10^6^	1986

**Table 2:** Quality parameters associated with 16 different geographically distributed urban lakes, China.

Urban Lakes	pH	TN	NO_3_^-^-N	NO_2_^-^-N	NH_4_^+^-N	TP	COD _Mn_	Fe	Mn	TOC
		(mg/L)								
TieXi (TX)	9.30 ± 0.24a	0.44 ± 0.06l	0.07 ± 0.01ef	0.01 ± 0.00b	0.03 ± 0.00j	0.01 ± 0.00h	5.79 ± 0.87c	0.03 ± 0.00bcd	0.01 ± 0.00bc	5.74 ± 0.28bc
XinHaiHu (XHH)	8.48 ± 0.06b	3.84 ± 0.33a	0.39 ± 0.09a	0.04 ± 0.03a	0.30 ± 0.05c	0.21 ± 0.01a	9.01 ± 0.31a	0.02 ± 0.00de	0.01 ± 0.00a	9.77 ± 0.71a
JinJi (JJ)	8.04 ± 0.14de	0.85 ± 0.26gh	0.06 ± 0.01fg	0.01 ± 0.00b	0.15 ± 0.07ef	0.05 ± 0.00cd	5.08 ± 0.10de	0.04 ± 0.01bc	0.01 ± 0.00a	5.69 ± 0.56d
ChangLe (CL)	7.72 ± 0.43de	0.57 ± 0.11jk	0.04 ± 0.01fg	0.04 ± 0.01a	0.10 ± 0.00ghi	0.06 ± 0.00b	4.70 ± 1.44f	0.04 ± 0.01a	0.00 ± 0.00c	1.09 ± 0.20f
XiangShan (XS)	7.45 ± 0.04gh	1.02 ± 0.07g	0.16 ± 0.03c	0.01 ± 0.00b	0.01 ± 0.00j	0.02 ± 0.00g	3.60 ± 0.25f	0.02 ± 0.00ef	0.01 ± 0.00ab	4.07 ± 0.55d
AiXi (AX)	7.40 ± 0.09gh	0.57 ± 0.16ij	0.12 ± 0.03d	0.01 ± 0.00b	0.13 ± 0.07e	0.03 ± 0.01f	5.13 ± 0.86cd	0.04 ± 0.012ab	0.01 ± 0.002a	5.61 ± 0.17bc
HuiLongShan (HLS)	7.88 ± 0.10def	0.52 ± 0.02f	0.06 ± 0.01fg	0.01 ± 0.00b	0.07 ± 0.01f	0.03 ± 0.01cd	4.43 ± 0.18ef	0.02 ± 0.00de	0.01 ± 0.00a	4.66 ± 0.59b
GaoTie (GT)	8.20 ± 0.19bc	0.35 ± 0.11kl	0.04 ± 0.02fg	0.01 ± 0.00b	0.07 ± 0.01i	0.05 ± 0.01ef	4.21 ± 0.11f	0.02 ± 0.00ef	0.01 ± 0.00a	3.78 ± 0.54a
JinSha (JS)	8.15 ± 0.19cd	2.08 ± 0.22m	0.04 ± 0.00fg	0.01 ± 0.00b	0.15 ± 0.03hi	0.05 ± 0.01cd	4.35 ± 0.80f	0.02 ± 0.01ef	0.01 ± 0.00a	9.33 ± 0.45a
XiLiu (XL)	7.79 ± 0.02ef	0.77 ± 0.10b	0.37 ± 0.06g	0.04 ± 0.01a	0.41 ± 0.01f	0.05 ± 0.01cd	4.42 ± 0.30ef	0.02 ± 0.00de	0.01 ± 0.00a	3.34 ± 0.19b
ZiZhuYuan (ZZY)	7.95 ± 0.02de	0.32 ± 0.03hi	0.22 ± 0.03a	0.01 ± 0.00b	0.11 ± 0.01b	0.05 ± 0.01cd	5.67 ± 0.80c	0.01 ± 0.01ef	0.01 ± 0.00a	3.17 ± 0.62e
GuiLong (GL)	7.27 ± 0.27hi	1.23 ± 0.40m	0.19 ± 0.23b	0.01 ± 0.00b	0.16 ± 0.03gh	0.05 ± 0.00d	6.23 ± 1.12cd	0.02 ± 0.01ced	0.01 ± 0.00a	5.77 ± 0.26b
ZhuZhai (ZZ)	7.28 ± 0.30i	0.70 ± 0.06ij	0.13 ± 0.05de	0.01 ± 0.00b	0.01 ± 0.00j	0.03 ± 0.01ef	8.35 ± 0.10a	0.01 ± 0.00efg	0.01 ± 0.002a	4.76 ± 0.14d
ZhongShan (ZS)	7.45 ± 0.03gh	1.37 ± 0.04e	0.05 ± 0.02fg	0.01 ± 0.00b	0.25 ± 0.00d	0.05 ± 0.00cd	7.1 ± 0.27b	0.02 ± 0.00ef	0.01 ± 0.002a	4.31 ± 0.41e
West Lake (WL)	7.94 ± 0.04de	1.05 ± 0.47d	0.15 ± 0.03cd	0.01 ± 0.00b	0.11 ± 0.01g	0.04 ± 0.00e	4.41 ± 0.81f	0.00 ± 0.00fg	0.01 ± 0.000a	2.82 ± 0.49e
YuNv (YN)	7.61 ± 0.05fg	2.08 ± 0.10c	0.11 ± 0.01de	0.10 ± 0.00b	0.50 ± 0.04a	0.06 ± 0.01b	8.33 ± 0.08a	0.00 ± 0.00g	0.01 ± 0.002a	5.59 ± 0.86b
One-way ANOVA	***	***	***	***	***	***	***	***	***	***

Values shown as means and standard deviations (*n* = 3). Different capital letter represents statistical significance. * *p* < 0.05, ** *p* < 0.01, and *** *p* < 0.001 represent statistical significance using one-way ANOVA followed by a post hoc Tukey’s honestly significant difference (HSD) test.

**Table 3:** The networks properties of algal communities at genus level.

Parameters	Number
Avg. weighted degree	0.548
Network diameter	7
Graph density	0.057
Modularity	1.446
Connected components	6
Avg. clustering coefficient	0.209
Avg. path length	3.231
Nodes	62
Edges	108

## Supplementary Materials

The following are available online at https://www.mdpi.com/1660-4601/17/3/1009/s1. The full name and abbreviations of urban lakes and water quality parameters associated with 16 different geographically distributed urban lakes, China (Table S1).
