# Supplementary material for: Geographical Patterns of Algal Communities Associated with Different Urban Lakes in China
Source: Int J Environ Res Public Health. 2020 Feb 5;17(3):1009. doi: 10.3390/ijerph17031009 (PMC7037785; doi:10.3390/ijerph17031009)
Supplement: Supplementary file 1 [file ijerph-17-01009-s001.pdf]

Supplementary Materials

Abbreviations

Table S1. The full name and abbreviations with urban lakes and water quality parameters associated with sixteen different geographically distributed urban lakes, China.

Heading	Full name	Abbreviations
Urban lakes	TieXi	TX
	XinHaiHu	XHH
	JinJi	JJ
	ChangLe	CL
	XiangShan	XS
	AiXi	AX
	HuiLongShan	HLS
	GaoTie	GT
	JinSha	JS
	XiLiu	XL
	ZiZhuYuan	ZZY
	GuiLong	GL
	ZhuZhai	ZZ
	ZhongShan	ZS
	West lake	WL
	Yunv	YN
Water quality parameters	Total nitrogen	TN
	Total phosphorus	TP
	Nitrate nitrogen	NO_3^--N
	Nitrite nitrogen	NO_2^--N
	Ammonia nitrogen	NH_4^+-N
	Total organic carbon	TOC
	Permanganate index	COD_{Mn}
